# Determinants Analysis Regarding Household Chemical Indoor Pollution

**DOI:** 10.3390/toxics11030264

**Published:** 2023-03-13

**Authors:** Paolo Montuori, Mariagiovanna Gioia, Michele Sorrentino, Fabiana Di Duca, Francesca Pennino, Giuseppe Messineo, Maria Luisa Maccauro, Simonetta Riello, Ugo Trama, Maria Triassi, Antonio Nardone

**Affiliations:** 1 Department of Public Health, University “Federico II”, Via Sergio Pansini n° 5, 80131 Naples, Italy; 2 General Directorate of Health, Campania Region, Centro Direzionale Is. C3, 80143 Naples, Italy

**Keywords:** indoor air quality, chemical contaminants, knowledge, attitude, practice, cross-sectional survey

## Abstract

Indoor household pollution is not yet sufficiently studied in the general population. Over 4 million people die prematurely every year due to air pollution in households. This study aimed to propose quantitative data research through the administration of a KAP (Knowledge, Attitudes, and Practices) Survey Questionnaire. This cross-sectional study administered questionnaires to adults from the metropolitan city of Naples (Italy). Three Multiple Linear Regression Analyses (MLRA) were developed, including Knowledge, Attitudes, and Behavior regarding household chemical air pollution and the related risks. One thousand six hundred seventy subjects received a questionnaire to be filled out and collected anonymously. The mean age of the sample was 44.68 years, ranging from 21–78 years. Most of the people interviewed (76.13%) had good attitudes toward house cleaning, and 56.69% stated paying attention to cleaning products. Results of the regression analysis indicated that positive attitudes were significantly higher among subjects who graduated, with older age, male and non-smokers, but they were correlated with lower knowledge. In conclusion, a behavioral and attitudinal program targeted those with knowledge, such as younger subjects with high educational levels, but do not engage in correct practices towards household indoor chemical pollution.

## 1. Introduction

More than 4 million people die prematurely every year due to household air pollution [1,2]. Elevated concentrations of indoor pollutants are not only associated with increased mortality but also with a range of harmful health effects, such as adverse pregnancy outcomes [3], chronic obstructive pulmonary disease [4], severe pneumonia, especially in childhood [5], lung cancer [6], cardiovascular diseases [7,8]. The greatest risk comes from long-term exposure, as 80–90% of a lifetime is spent in confined spaces which may increase due to cumulative lifetime exposures [9,10,11].

Indoor air pollution is a significant public health issue, caused by various substances found in common household items and influenced by common indoor activities, such as heating, cooking, and the use of cleaning products, as well as behavioral practices like smoking, vaping, burning candles or incense [12,13,14,15]. Moreover, many of these pollutants can cause secondary reactions producing additional highly reactive and harmful substances [16].

Public policy is a crucial tool in reducing air pollution and improving air quality and people’s health. Since 1990, measures designed to curb air pollution have prevented approximately 600,000 premature deaths annually [17]. The Control Action Plan introduced a decade ago has already prevented 15,822 associated morbidities in 2017 [18]. However, these policies have mostly focused on outdoor environments, ignoring indoor spaces where people spend most of their time [19].

In fact, individuals can play a crucial role in reducing their exposure to indoor air pollution, as their behavior significantly impacts the indoor environment [20]. By following specific yet reasonable behaviors, individuals can reduce the risks associated with indoor air pollution. Such practices include ensuring adequate ventilation, maintaining combustion appliances, limiting exposure to volatile organic compounds, and reducing smoking [21]. Improving ventilation rates in households by opening windows or using ventilation fans can lead to a reduction in emissions from human activities, thereby improving indoor air quality [22,23]. Additionally, higher ventilation rates have been linked to improved health outcomes [24].

A recent study assessed the dependence of community knowledge and attitude with socio-demographic factors and the dependence of the behaviors with knowledge, attitude, and socio-demographic factors using community KAB towards IAQ, revealing lower levels of knowledge and behaviors towards IAQ and moderate levels of attitude within the study population [25]. Daniel et al. described the perceptions, knowledge, and practices of adults concerning indoor environmental pollution, evidencing that well-integrated practices were not related to knowledge, level of education, or perceptions but rather to the responsibility of having a child and that implementation of less well-followed practices would be improved by better knowledge/information and a change in perceptions [26]. In 2018, Al-Khamees examined the knowledge, attitudes, and practices toward indoor pollution at Kuwait University, demonstrating poor knowledge regarding indoor pollution among university students and teachers [27]. Moreover, some papers focused their research only on certain types of pollutants, such as Adeolu et al., that conducted a study on knowledge and attitudes towards lead exposure in Nigeria [28], or over radon, a typical yet specific pollutant for some households, in a KAP model conducted in 2018 in a rural environment by Neri et al. [29].

For those reasons, the present study aims to propose quantitative research of data through the administration of a KAP (Knowledge, Attitudes, and Practices) Survey Questionnaire and the statistical analysis of the information collected towards household chemical air pollution in a population of a large metropolitan area to understand this phenomenon in order to collect data to develop specific and tailored educational programs.

## 2. Materials and Methods

### 2.1. Setting and Sample

This cross-sectional study was conducted by administering questionnaires to adults from the metropolitan city of Naples (Italy), with a population of 909,048 [30]. The study was conducted from the beginning of January 2022 to the end of September 2022. Subjects were selected to participate in the study using a snowballing sampling method among universities, working places, and community centers. The inclusion criteria in the study required that participants were 18 and older and residing in the metropolitan area of Naples. The required sample size was calculated using Slovin’s formula to obtain a representative sample within a margin of error of 3%, and a confidence interval of 95%, determining a final number of subjects to be recruited of 1523. Finally, after accounting for a 30% non-response rate, the estimated total sample size was 1066.

### 2.2. Procedures

During the study period, experienced interviewers submitted to participants the questionnaire from Monday to Friday between 10:00 a.m. and 8:00 p.m. to avoid over-sampling non-working individuals. The interviewers, at the beginning of the submission, stated that they were conducting a study on behalf of the University of the studies of Naples “Federico II”, giving information to the participants about the nature and scope of the research, the methodology, that their participation was on a voluntary basis, that all the collected information would be processed anonymously and confidentially, and that they could end their participation at any time without disclosing a reason. Verbal informed consent was obtained prior to progressing with the interview. No incentive for participation or survey completion was provided. The present study conformed with the Declaration of Helsinki, and ethical clearance was obtained according to local legislation.

### 2.3. Data Collection

The questionnaire was developed through the meeting of a large commission of physicians, chemists, and biologists. Questions considered inappropriate or not useful for the study objectives were either removed or replaced. Before the commencement of the data collection, a pilot study was performed on 10 individuals in order to test the participants’ understanding of the questionnaire items, the results of which were not taken into consideration for the study. The first section of the questionnaire assessed participants’ socio-demographic characteristics and other health-related information, including gender, age, marital status, level of education, occupation, partner’s occupation, and number of children. The second section investigated knowledge, attitudes, and behaviors concerning household chemical air pollution for a total of 36 questions. Knowledge and attitudes were assessed on a three-point Likert scale with options for “agree”, “uncertain”, and “disagree”, while inquiries regarding behaviors were in a four-answer format of “never”, “sometimes”, “often”, and “yes/always”.

### 2.4. Statistical Analysis

Data reported by the study were analyzed using STATA MP v14.0 statistical software program (College Station, TX, USA). The analysis was carried out in two steps. First, a descriptive statistic was employed to sum up the basic information of the statistical units; then, a Multiple Linear Regression Analysis (MLRA) was performed, as previously extensively explained [31,32,33]. Briefly, three MLRA were developed, including the variables potentially associated with the following outcomes of interest:

(1) Knowledge regarding household chemical air pollution and the related risks (Model 1);
(2) Attitudes toward household chemical air pollution (Model 2);
(3) Behavior related to household chemical air pollution (Model 3).

Knowledge, Attitudes, and Behaviors, as dependent variables, were acquired by adding the results of the respective question scores (questions with inverse answers have been coded inversely). The independent variables were included in all models: sex (1 = male, 2 = female); age, in years; education level (1 = primary school, 2 = middle school, 3 = high school, 4 = university degree); marital status (1 = Single; 2 = In a relationship); smoking habits (1 = smoker, 2 = non-smoker); having children (1 = Yes; 2 = No). In Model 2, Knowledge was added to the independent variables, and in Model 3, both Knowledge and Attitudes were included in the independent variables. Attitudes and Knowledge were analyzed as indexes rather than scales; thus, each observed variable (A1, …, A10 and K1, …, K11) is presumed to cause the latent variables associated (Attitude and Knowledge). In other words, the relationship between observed and latent variables is formative. Therefore, inter-observed variables correlations are not required. On the contrary, the relationship between the observed variables (B1, …, B11) and latent variable Behavior could be considered reflective (Cronbach’s alpha = 0.825). All statistical tests were two-tailed, and results were statistically significant if the p-values were less than or equal to 0.05.

## 3. Results and Discussion

During the administration period, 1670 subjects were recruited to participate in the study and received a questionnaire to be filled out and collected anonymously. Among those, 1332 questionnaires were filled and returned with a response rate of 79.76%, slightly more than expected (70%) and calculated. Characteristics of the sample are described in Table 1. Regarding gender, 677 were male (50.83%) and 655 females (49.17%). The mean age of the study population was 44.68, ranging from 21–78 years. Educational levels were distributed as 51 subjects (3.83%) declaring an elementary school license, 332 (24.92%) middle school license, 506 (37.99%) responding to having a high school diploma, and 443 (33.26%) graduated with a university degree. Responding about their marital status, 379 respondents (28.45%) declared themselves to be single, and 953 were in a relationship; in addition, 675 of them stated to have at least a son, while 675 had none. Finally, more than half of the population surveyed (59.68%) said they did not smoke. Therefore, this sample can be considered representative of a standard European population in size and frequency of main demographic characteristics [34].

Respondent’s knowledge about household indoor pollution is presented in Table 2. Most of the people interviewed knew that the chemical pollution of the air in the household environment is more than that of the outdoors (74.25%). The 0.23% of the sample had not answered to K1 question. Only 38.29% of the population knew that gas stoves contribute to household pollution, while 36.94% did not. Half of the sample (50.00%) believed that plants at night release substances dangerous to health. Regarding smoke, most respondents (52.48%) knew where second-hand smoke comes from, while less than half (40.24%) disagreed that thirdhand smoke is less toxic than secondhand smoke. In addition, 65.24% of the population agreed that inadequate ventilation causes more than 50% of the chemical pollution of the air in a domestic environment. Moreover, a high percentage of the respondents knew that carbon monoxide is the main household pollutant (68.17%), and that formaldehyde is a household chemical pollutant (71.02%). However, only 46.16% of the population knew that formaldehyde is classified as a carcinogen. Half of the sample, 50.83%, knew what Sick Building Syndrome is, and only 21.40% were aware that currently, there are no laws governing the pollution of the domestic environment. Furthermore, with a mean score of 69.19%, the population showed a good knowledge regarding household chemical pollution. Similar results were evidenced in 2020 in France by Daniel et al., where a population of 554 adults totalized a mean score of 68.19% [26]. However, other studies revealed lower levels of knowledge, as evidenced in 2020 by Muro et al. in a study conducted in Nairobi County over a sample of 393 subjects, which indicated a low knowledge level on indoor air pollution with an average score of 38.5% and, previously, by Al-Khamees in 2018, in Kuwait, which demonstrated that the respondents had a low knowledge level on indoor air pollution at 41.47% [27,35]. These differences can be justified considering the diversity of the populations sampled for the study and the significant difference in the distribution of educational levels between them [36].

Table 3 describes attitudes towards household chemical indoor pollution. The vast majority of the respondents (90.90%) agreed with the good habit of opening the windows, in agreement with Amegah et al. [3], who observed that even with the air conditioner turned on (52.48%), spending time in home microenvironments may not offer sufficient protection from fine ambient aerosol particles (PM_2.5_) [37] and that risk factors for fine particles (PM_2.5_) are greater than for coarse particles (PM_10_) [38]. Thus, 45.95% of the population deemed it necessary to ventilate the house in winter. The majority of the sample (76.13%) had a good attitude toward house cleaning, and 56.76% of the respondents stated that it was important to pay attention to the use of cleaning products. In addition, almost half of the sample (48.87%) believe it is convenient to use spray deodorant, and 41.67% think lighting candles at home is relaxing. Concerning the last two attitudes, an agreement was noted with the results obtained by Al-Khamees, who evidenced similar results [27]. More than half (56.53%) believed having plants in the house is nice. Unfortunately, only 33.56% thought that induction stoves are more comfortable than gas ones, and 64.19% believed that a fireplace improves the house. Roughly half of the sample (48.49%) deemed smoking on the sofa as not relaxing.

The behaviors of respondents are listed in Table 4. 55.18% of the sample replied that they were attentive to the ventilation of their own house, but only 10.81% claimed to use air purifiers. Regarding gas stoves, almost half of the population (43.47%) use them all the time. Despite the extensive use of gas stoves, only 29.28% operate the hood in the kitchen while cooking food. It has also been noted that there is still a large use of pellet or gas stoves (54.50%), while there is more focus on the use of filters for heating/conditioning systems (47.07%) and checking them (49.55%). Fortunately, the use of insecticides is not widespread, as well as that of air fresheners (23.42%). Of all the behaviors, the most comforting fact comes from smoking, which is never practiced at home, from 54.50% of the population about traditional cigarettes and 61.49% for heated tobacco cigarettes. A high percentage of incorrect behaviors were encountered in the sample, meanly 64.59% for men and 64.69% for women. Those scores were slightly higher than Al-Khamees et al., which were 51.0% for men and 53.5% for women [27]. Also, Daniel et al. found out that certain practices were not well followed by less than 60% of participants [26]. The reason why the results revealed high percentages of some incorrect behaviors rather than others is probably that these are actions performed repeatedly in daily life, and many of them become incorrect habits fueled by poor knowledge and understanding of household air pollution.

Table 5 illustrates the results of linear multiple regression in three models. Model I, Knowledge, as a dependent variable, was correlated with age and education, evidenced as younger subjects had a better overall consciousness of household chemical pollution. These findings agreed with a previous study by Unni in Singapore in 2022, which evidenced a decreasing level of knowledge within the elder population, and with another KAP study carried out over 1604 subjects resident in Ningbo, China, that showed a similar trend of declining levels of knowledge [25,39]. Furthermore, the findings of this investigation are consistent with Jin et al., who analyzed knowledge regarding Secondhand Smoke Exposure and assessed that about 60% of people aged between 15 and 34 had better knowledge of the harmful effects of smoking than people aged 60 [40]. Therefore, since, to the best of our knowledge in literature, no other paper has evidenced a higher knowledge regarding indoor pollution in the elder population, this result may suggest a more pronounced awareness of pollution in younger subjects, as clarified by Chin et al. in 2019 [41]. The second evidence of this MLRA was the statistically significant relation between knowledge regarding indoor air pollution and education. In particular, higher knowledge levels were found in subjects with higher education levels. This evidence is widely expected and confirmed by Kaur et al., who stated that, among a sample of urban homemakers in Ludhiana (India), urban respondents with a higher education level were more conscious of environmental concerns than their own rural counterparts [42]. In addition, Daniel et al. in 2020 found that a higher level of education was also associated with a higher knowledge score in a population of adults between 18 and 45 years in Brittany (France) [26]. These results are not surprising since educational level is widely reported in the literature as a predictor of pollution-related knowledge [43,44,45]. Moreover, in a cross-sectional study conducted in Italy over 15 universities, the perception of environmental health risks was positively associated with increasing years of attending classes, such as the interest in searching for different sources of information [46].

Model II uses Attitudes as a dependent variable assessing a positive correlation, statistically significant, with age, gender, education, smoking habits, and knowledge. In particular, the regression analysis results indicated that positive attitudes were significantly higher among subjects who graduated, with older age, male and non-smokers, but they were correlated with lower knowledge. Regarding the correlation between age and attitude, as found in the present study, the literature reports the study conducted by Unni et al., in 2022, on household residents in Singapore, which evidenced that older residents had a higher attitude score than newer counterparts [25]. This result is widely expected as it has been stated that younger persons have a significantly worse perception of air pollution [47] and of activities that may reduce related health harnesses, whereas elder subjects are more aware of the risks [48]. Also, with reference to the between attitudes and gender, the results of the MLRA highlighted that females had a better overall score in attitude, according to the study by Al-Khamees et al., which, in a sample of students and teachers at Kuwait University, found a significantly better attitude in females, stating that such correlation can be explained as women are more often involved in polluting activities and tend to be less on guard regarding the risks connected [27]. Moreover, the evidence related to positive attitudes and respondents with higher education was confirmed in the study by Unni et al., that assessed community levels of Knowledge, Attitude, and Behavior (KAB) towards indoor air quality in randomly selected adults in Singapore: those who were higher skilled had comparatively higher attitude scores [25]. This result also agreed with Egondi et al., who in 2013 reported the association between attitude and educational levels, and Liu et al., who stated that a lower consciousness of air pollution and health effects was associated with a low educational level [49,50]. Furthermore, a recent cross-sectional study carried out in Lebanon over 2623 participants assessed that attitude towards cumulative effects of smoking, therefore also related to indoor air pollution, was significantly higher in nonsmoker subjects [51]. In addition, Al-Haqwi reported that non-smokers among a population of students had more willingness to act against polluting activities and therefore had better attitudes [52]. Also, the surprising relationship between attitudes and lower knowledge scores was confirmed in the aforementioned study by Unni et al., who assessed the same correlation [25]. Since, as aforementioned, knowledge is negatively related to behaviors, another educational program has to be implemented, in this case, designed to improve knowledge targeted to categories of people who allegedly already have positive attitudes and correct behaviors with the aim to reinforce their habits and improve their already good practices such as subjects involved in a relationship, with high educational levels and, non-smokers.

In conclusion, a behavioral program targeted those with knowledge, such as younger subjects with high educational levels, but do not engage in the correct practice toward indoor chemical pollution.

Model III displays that practices regarding household air pollution were statistically significant and correlated to education, smoking habits, knowledge, and attitudes. It has also been noted that there was a positive correlation between correct behaviors and marital status. In relation to the latter, a cross-sectional study conducted in Nairobi (Africa) on over 5317 individuals aged 35+ showed that marital status was not associated with improved behavior leading to better air quality [49]. Moreover, Kim et al. indicated that married subjects had better attitudes toward air pollution, but an explanation was not provided [53]. Therefore, the literature suggests that people involved in a relationship are usually more concerned about environmental pollution because their partner synergically influences them [54]. The relation between positive practices and education level might appear obvious; however, some doubts arise from the review by Maung et al. about indoor air pollution, which highlighted how human activities, behaviors, and education level are associated with personal exposure to air pollutants [55]. Again, as expected, teachers had a higher level of knowledge than students, which was reflected in their use of less polluting behaviors [27].

The results of the MLRA also evidenced the relationship between behavior and non-smoking. However, although widely expected, the literature does not define this correlation well. So far, only one previous study, conducted on householders in the USA during 2010–2011, evidenced that subjects without smoking habits also had other behaviors correlated to reduced air pollution [56]. Therefore, indoor pollution-related behaviors and air pollution, in general, may be affected by having or not smoking habits. Besides, in this study, a relation between positive behavior and negative knowledge was found, also stated by Unni et al. [25].

Inherently, a review carried out by Barnes, comprehending data from several studies, defined the limited effectiveness of education in improving behaviors concerning indoor air pollution [57]. On the other hand, the study by Daniel et al. indicated an association between high knowledge levels and behavior scores [26]. This could explain the correlation found in our study related to positive behaviors and negative knowledge, unlike many other studies on indoor air pollution, which used the KAB model and found that higher knowledge levels for respondents towards IAQ were associated with significantly higher behavior scores [25,26]. Another important correlation found in Model III was between respondents with higher behavior scores and high attitude scores, in agreement with Unni et al. [25] and also consistent with previous literature, as pointed out by Pampel et al. in 2010, which demonstrated that subjects with better attitude also had better behaviors [58]. This relationship pointed out the dominant role of attitude in forming correct behaviors related to indoor pollution and led us to suggest that an educational program designed to improve attitude is mandatory to improve behaviors in the population. Moreover, it is necessary to organize a training program for those who demonstrate the worst behaviors, such as singles, smokers, and less-educated subjects, to improve their practices and reduce the quantity of indoor pollutants they are exposed to and, therefore, the risks associated with it.

## 4. Conclusions

In summary, as shown in this study, indoor household pollution is a phenomenon not yet sufficiently studied in the general population. Behaviors intended to reduce indoor pollution are difficult to practice, although the sample has a good knowledge of the harms resulting from some habits. Therefore, it is necessary to organize training programs for people with the worst behavior, such as singles, smokers, and less-educated people, to improve their practice and reduce the amount of pollutants they are exposed to within the house and, therefore, the risks associated with it. Since knowledge is negatively related to behavior and attitude, it is necessary to implement another educational program in this case to improve knowledge of a category of people who allegedly already have positive attitudes and correct behaviors in order to strengthen their habits and improve their good practices, such as subjects involved in high-level relationships and non-smokers. In conclusion, a behavior and attitude correction program is aimed at those with knowledge, such as young people with high education levels, but does not put proper practices for household indoor chemical pollution in practice.

## Figures and Tables

**Table 1:** Study population characteristics.

Study Population	N (1332)	Percentage
**Sex**		
**Male**	677	50.83
**Female**	655	49.17
**Age**		
**<30**	327	24.55
**31–35**	209	15.69
**36–40**	91	6.83
**41–45**	93	6.98
**46–50**	105	7.88
**>51**	507	38.06
**Education**		
**Primary school**	51	3.83
**Middle school**	332	24.92
**High school**	506	37.99
**University Degree**	443	33.26
**Children**		
**Yes**	675	50.68
**No**	657	49.32
**Smoking habits**		
**Yes**	537	40.32
**No**	795	59.68
**Marital Status**		
**Single**	379	28.45
**In a relationship**	953	71.55

**Table 2:** Knowledge of respondents regarding household chemical indoor pollution.

N.	Statement (Variables)	Agree (%)	Uncertain (%)	Disagree (%)
**K1**	The chemical pollution of the air in the household environment is less than that of outdoors *	6.53	18.99	74.25
**K2**	Gas stoves contribute to household pollution	38.29	24.77	36.94
**K3**	Plants at night release substances dangerous to health	50.00	35.36	14.64
**K4**	Secondhand smoke comes from the smoke exhaled by a smoker	52.48	24.55	22.97
**K5**	Thirdhand smoke derives from the toxic substances of the smoke deposited in the environment	40.24	20.35	39.41
**K6**	Thirdhand smoke is less toxic than secondhand smoke	34.68	25.80	40.24
**K7**	Inadequate ventilation causes more than 50% of the chemical pollution of the air in the domestic environment	65.24	29.13	5.63
**K8**	The main household pollutant is carbon monoxide	68.17	24.17	7.66
**K9**	Formaldehyde is one of the household chemical pollutants	71.02	25.38	7.06
**K10**	Formaldehyde is a certain carcinogen	46.62	42.79	10.59
**K11**	The Sick Building Syndrome is a condition in which the occupants of a building show a series of symptoms and pathologies without specific causes	50.83	30.93	18.24
**K12**	There are laws governing the pollution of domestic environments	47.90	30.71	21.40

* 0.23% of the sample did not respond to the K1 question.

**Table 3:** The attitude of respondents toward household chemical indoor pollution.

N.	Statement (Variables)	Agree (%)	Uncertain (%)	Disagree (%)
**A1**	Opening windows is a good habit	90.90	8.78	1.13
**A2**	It is necessary to open the windows even with the air conditioner on	52.48	22.52	25.00
**A3**	In winter, it is still necessary to ventilate the house several times a day	45.95	20.05	34.00
**A4**	House cleaning is a waste of time	13.96	9.91	76.13
**A5**	A cleaning product is as good as another	20.72	22.52	56.76
**A6**	It is convenient to use spray deodorant	48.87	28.15	22.97
**A7**	It is nice to have plants in the house	56.53	19.82	23.65
**A8**	Lightning candles at home is relaxing	41.67	22.75	35.59
**A9**	Induction stoves are no more comfortable than the gas ones	44.59	21.85	33.56
**A10**	A fireplace graces the house	64.19	18.02	17.79
**A11**	Smoking on the sofa is relaxing	38.29	13.29	48.49

**Table 4:** Behaviors of respondents concerning household chemical indoor pollution.

N.	Questions	Yes/Always (%)	Often (%)	Sometimes (%)	Never (%)
**B1**	Do you ventilate your home?	55.18	27.70	6.98	10.14
**B2**	Do you use air purifiers?	10.81	18.69	30.63	39.86
**B3**	Do you use gas stoves?	43.47	15.09	12.16	29.28
**B4**	Do you operate the hood in the kitchen?	29.28	15.99	18.47	36.26
**B5**	Do you use gas and/or a pellet heater?	54.50	12.61	18.92	13.96
**B6**	Do you use filters for heating/air conditioning systems?	47.07	7.43	17.34	28.15
**B7**	Do you periodically check the heating, air conditioning, and ventilation systems?	49.55	10.36	12.84	27.25
**B8**	Do you use insecticides at home?	16.67	16.22	11.71	55.41
**B9**	Do you use air fresheners?	23.42	14.19	5.63	56.76
**B10**	Do you wash curtains and carpets?	18.24	10.81	10.59	60.36
**B11**	Do you decorate your home with plants?	45.95	18.47	18.24	17.34
**B12**	Do you smoke traditional cigarettes in your home?	22.75	9.46	13.29	54.50
**B13**	Do you smoke heated tobacco cigarettes in your home?	14.64	8.11	15.77	61.49

**Table 5:** Results of the linear multiple regression analysis (MLRA).

	Coefficients Not Standardized		Coefficients Standardized			
	b	Standard Error	t	95% Conf. Interval		*p*-Value
**Model I—Dependent variable: Knowledge**						
*Prob > F = 0.000*	*R-squared = 0.0323*			*Root MSE = 3.929*		
Age	−0.022	0.009	−2.33	−0.041	−0.003	0.020
Sex	−0.175	0.216	−0.081	−0.599	0.025	0.416
Marital status	−0.218	0.274	0.80	−0.319	0.755	0.425
Children	0.082	0.306	0.27	−0.517	0.681	0.681
Education	0.544	0.140	3.88	0.269	0.818	0.000
Smoking habits	0.004	0.220	0.02	−0.427	0.435	0.099
**Model II—Dependent variable: Attitudes**						
*Prob > F = 0.000*	*R−squared = 0.0532*			*Root MSE = 3.386*		
Age	0.021	0.008	2.58	0.005	0.037	0.010
Sex	−0.396	0.186	−2.13	−0.761	−0.032	0.033
Marital status	−0.455	0.236	−1.93	−0.918	0.007	0.054
Children	0.238	0.263	−0.090	0.278	0.754	0.366
Education	0.597	0.121	4.92	0.359	0.835	0.000
Smoking habits	0.862	0.189	4.55	0.490	1.23	0.000
Knowledge	−0.1199	0.024	−5.08	−0.166	−0.074	0.000
**Model III—Dependent variable: Behavior**						
*Prob > F = 0.000*	*R−squared = 0.1617*			*Root MSE = 6.901*		
Age	0.004	0.017	0.22	−0.029	0.037	0.825
Sex	−0.013	0.379	−0.03	−0.757	0.731	0.973
Marital status	1.05	0.482	2.19	0.109	1.99	0.029
Children	−0.262	0.537	−0.49	−1.31	0.790	0.626
Education	1.39	0.249	5.59	0.905	1.88	0.000
Smoking habits	1.77	0.389	4.56	1.01	2.54	0.000
Knowledge	−0.415	−0.048	−8.55	−0.510	−0.319	0.000
Attitude	0.516	0.056	9.24	0.406	0.625	0.000

## Data Availability

The data that support the findings of this study are available upon reasonable request from the corresponding author. The data are not publicly available due to privacy or ethical restrictions.
